# Role of oxidative stress and intracellular glutathione in the sensitivity to apoptosis induced by proteasome inhibitor in thyroid cancer cells

**DOI:** 10.1186/1471-2407-9-56

**Published:** 2009-02-16

**Authors:** Zhen-Xian Du, Hai-Yan Zhang, Xin Meng, Yifu Guan, Hua-Qin Wang

**Affiliations:** 1 Department of Endocrinology and Metabolism, the 1st Affiliated Hospital, China Medical University, Shenyang, PR China; 2 Department of Geriatrics, the 1st Affiliated Hospital, China Medical University, Shenyang, PR China; 3 Department of Biochemistry and Molecular Biology, China Medical University, Shenyang, PR China

## Abstract

**Background:**

The proteasome inhibitor bortezomib has shown impressive clinical activity alone and in combination with conventional and other novel agents for the treatment of multiple myeloma (MM) and some solid cancers. Although bortezomib is known to be a selective proteasome inhibitor, the downstream mechanisms of cytotoxicity and drug resistance are poorly understood.

**Methods:**

Proteasome activity, intracellular glutathione (GSH) and ROS levels, as well as activities of GSH synthesis enzymes were measured using spectrophotometric methods. Cell death was analyzed using flow cytometry and caspase activity assay. The expression level of GSH synthesis enzymes were measured using real-time RT-PCR.

**Results:**

At concentrations that effectively inhibited proteasome activity, bortezomib induced apoptosis in FRO cells, but not in ARO cells. Bortezomib elevated the amount of glutathione (GSH) and the treatment with bortezomib increased the level of mRNA for GCL, a rate-limiting enzyme in glutathione synthesis. Furthermore, depletion of GSH increases apoptosis induced by bortezomib, in contrast, repletion of GSH decreases bortezomib-mediated cell death.

**Conclusion:**

GSH protects cells from proteasome inhibition-induced oxidative stress and glutathione-dependent redox system might play an important role in the sensitivity to proteasome inhibition-induced apoptosis.

## Background

Protein degradation is crucial for controlling the availability of regulatory proteins in the cell. The ubiquitin-proteasome system (UPS) represents the major degradation pathway for proteins involved in the regulation of cell survival, proliferation, apoptosis and other critical cellular functions [1]. UPS also has a critical role in the selective removal of mutant, damaged, and misfolded proteins. Based on these unique properties of the UPS, the proteasome pathway has recently emerged as an attractive target for the development of anti-cancer agents. Proteasome inhibitors represent a diverse group of agents that target the 20S proteasome, a component of the UPS that is responsible for the degradation of unwanted cellular proteins [2]. Although the mechanism by which proteasome inhibitors kill neoplastic cells is not known with certainty, it has been shown that proteasome inhibitors interfere with key steps of tumor cell regulation, leading to a block of proliferation and death of neoplastic cells both *in vitro *and *in vivo*, thus emerging as a new class of anticancer drugs. Experimental and clinical support for this therapeutic strategy has been provided by bortezomib [3,4].

The involvement of oxidative stress is supported by emerging studies showing that reactive oxygen species (ROS) generation plays a critical role in the initiation of the bortezomib-induced apoptotic cascade [5]. Oxidative stress is a complex and dynamic situation characterized by an imbalance between the productions of ROS and the availability and action of antioxidants. Oxidative stress has traditionally been considered in negative terms due to its association with macromolecular damage and triggering cell death through the effects of oxidants on signal transduction pathways, including the activation of sphingomyelinase, caspases and cathepsin D [6-10]. Generation of ROS is now considered to be the early and critical events for the initiation of bortezomib-induced apoptotic signaling in some human cancer cell lines [5,11-13]. One of the major mechanisms by which cells protect themselves against oxidative stress is upregulation of a wide range of antioxidant genes. Among intracellular antioxidant molecules, reduced glutathione (GSH) is the most abundant intracellular non-protein thiol in cells. By keeping the cellular environment in a reduced state, GSH functions in the removal of potentially toxic electrophiles and metals, thereby protecting cells from toxic oxygen products [14]. Furthermore, GSH exhibits a large panel of actions in controlling gene expression, apoptosis mechanisms, or membrane transport [15]. Therefore, cells tightly regulate the synthesis, utilization and export of GSH. L-S,R-buthionine sulfoximine (BSO) is a potent specific inhibitor of γ-glutamylcysteine synthetase, the rate-limiting enzyme in GSH biosynthesis, and has been used to deplete intracellular GSH and to reverse drug resistance in tumor cells [16]. GSH has for long been known a chemoresistance factor in cancer cells [17].

Several studies have suggested that UPS is involved in the regulation of anti-oxidants, such as catalase, heme oxygenase-1 (HO-1), copper/zinc-superoxide dismutase and γ-GCS [18-21]. The relationship between intracellular GSH level and proteasome inhibition-induced apoptosis still remains to be defined. To establish the contribution of ROS to bortezomib-induced thyroid cancer cell death, we performed a comparative kinetic analysis of ROS levels in ARO and FRO cells. In addition, we modulated the intracellular GSH level by depletion/repletion to study the role of this variable in cell sensitivity to treatment with bortezomib in human thyroid cancer cells. We found that ROS generation played a critical role in bortezomib-mediated apoptosis in thyroid cancer cells. Our results indicate that GSH depletion could be useful to increase the therapeutic efficacy of thyroid cancer treatment by bortezomib.

## Methods

### Multiple undifferentiated thyroid cancer cell lines

The ARO81-1 (simply ARO) and FRO82-1 (simply FRO) cell lines were initially obtained from Dr. James A. Fagin (University of Cincinnati College of Medicine, Cincinnati, OH) and provided to us by Dr. Shunichi Yamashita (Nagasaki University Graduate School of Biomedical Sciences, Japan). KTC2 cell line was generously provided by Dr. Junichi Kurebayashi (Kawasaki Medical School, Japan). 8305C cells were obtained from the European Collection of Animal Cell Cultures. All cell lines were maintained in RMPI 1640 (Sigma-Aldrich, Saint Louis, MO) supplemented with 10% fetal bovine serum (FBS, Sigma-Aldrich, Saint Louis, MO).

### Chemicals

Bortezomib was obtained from Millennium Pharmaceuticals Inc. (Cambridge, MA) and was dissolved in dimethyl sulfoxide (DMSO) as a stock solution. Specific GSH synthesis inhibitor (BSO) and glutathione were from Sigma-Aldrich. N-Acetyl-L-cysteine (L-NAC; Calbiochem, San Diego, CA) was prepared in sterile water immediately before use.

### 20S proteasome activity assay

Cytosolic extracts (without protease inhibitors) were used to measure proteasome activity using 20S proteasome assay kit (Chemicon International, Temecula, CA) following the manufacturer's instructions. The assay is based on detection of the fluorophore 7-amino-4-methylcoumarin (AMC) after cleavage from the labeled substrate LLVY-AMC. Levels of released AMC were measured using an excitation wavelength of 380 nm and an emission wavelength of 460 nm with an automatic multi-well plate reader. The relative activity was standardized by protein concentration, determined using Coomassie Protein Assay Reagent (Pierce, Rockford, IL).

### Detection of cell death

For cell death assays, cells were washed twice in phosphate-buffered saline and then stained with Annexin V-FITC (Biovision, Mountainview, CA) and propidium iodide (PI, Sigma-Aldrich) according to the manufacturer's instructions. After staining with annexin V-FITC and PI, samples were analyzed by fluorescence-activated cell scanner (FACScan) flow cytometer (Becton Dickinson, Franklin Lakes, NJ).

### Caspase-3 activity assay

For caspases-3 enzymatic assays, 50 *μ*g of whole cell extract were added to reaction buffer containing 25 mM HEPES (pH 7.5), 4 mM CHAPS, 1 mM DTT, 1 mM PMSF, 2 *μ*g/ml aprotinin, 1 *μ*g/ml leupeptin, and 2 *μ*g/ml pepstatin, to achieve a total reaction volume of 500 *μ*l. Ac-DEVD-AMC (Ac-Asp-Glu-Val-Asp-7-amino-4-methylcoumarin; Alexis Biochemicals, San Diego, CA) was added to the mixture at a concentration of 100 *μ*M and incubated for 1 hour at 37°C. Cleavage of the substrate was measured by fluorescence spectrometer (HTS 7000, Perkin Elmer, Boston, MA) using an excitation and emission wavelength of 360 nm and 465 nm, respectively. The activities were expressed as fluorescence increase per *μ*g of protein.

### Measurement of intracellular ROS levels

The average level of intracellular ROS in thyroid cancer cells was evaluated in cells loaded with the redox-sensitive dye DCFH-DA (Molecular Probes, OR). Cells were washed twice in a phosphate-buffered saline (PBS) and stained in the dark for 30 min with 20 μM DCFH-DA and harvested. Cells were dissolved with 1% Triton X-100, and fluorescence was measured at an excitation wavelength of 485 nm and an emission wavelength using a fluorescence spectrometer (HTS 7000, Perkin Elmer, Boston, MA). A duplicate culture with the same treatments was used to determine the total protein levels. The ROS levels were expressed as arbitrary unit/mg protein, then as the percentage of control.

### Measurement of glutathione levels

Cells were scraped and collected by centrifugation. The cell pellet was resuspended in 5% metaphosphoric acid (Sigma-Aldrich) and supernatant was collected for determining total glutathione and oxidized glutathione (GSSG) concentrations using a commercial kit according to the manufacturer's protocol (glutathione assay kit; Trevigen Inc., Gaithersburg, MD). Reduced glutathione (GSH) concentration was obtained by subtracting the oxidized from the total concentration. A duplicate culture with the same treatments was used to determine the total protein levels. The GSH or GSSG levels were expressed as nmol/mg of protein and then as the percentage of control.

### GSH synthesis enzyme activity assays

Cells were collected, homogenized, and centrifuged at 12000 × g for 30 min. The glutamate cysteine ligase (GCL) and glutathione synthetase (GS) enzyme activities were determined by the formation of ADP using a coupled assay with pyruvate kinase and lactate dehydrogenase. The GCL reaction mixture (final volume, 200 μl) contained 100 mM Tris-HCl buffer (pH 8.2), 20 mM MgCl_2_, 150 mM KCl, 10 mM L-glutamate, 10 mM L-cysteine, 5 mM ATP, 2 mM EDTA, 0.2 mM NADH, 2 mM phosphoenolpyruvate, 2 units of pyruvate kinase and 2 units of lactate dehydrogenase. The GS reaction mixture contained 100 mM Tris-HCl buffer (pH 8.2), 20 mM MgCl_2_, 50 mM KCl, 10 mM ATP, 2 mM EDTA, 5 mM glycine, 5 mM L-γ-glutamyl-L-α-aminobutyrate, 0.2 mM NADH, 0.5 mM phosphoenolpyruvate, 2 units of pyruvate kinase, and 2 units of lactate dehydrogenase. The reaction was initiated by adding 100 μg of protein extract, and the absorbance at 340 nm was monitored. The specific activity is expressed as units/mg of protein and then as a percentage of control.

### Real time RT-PCR analysis

Real time RT-PCR was performed to determine the mRNA levels of GCL and GS. Total RNA was isolated from cultures using Trizol reagent (Invitrogen, Carlsbad, CA) according to the manufacturer's protocol. RT and real-time PCR was performed as previously described [22]. For GCLC, the forward primer was 5'-CAAGGACGTTCTCAAGTGGG-3' and the reverse was 5'-CATACTCTGGTCTCCAAAGG-3'. For GS, the forward primer was 5'-ATGGACATGGTGAGCAACCAG-3' and reverse was 5'-CTTGACTCCAGCATACAAGC-3'. For β-actin, the forward primer was 5'-GAGACCTTCAACACCCCAGCC-3' and the reverse was 5'-GGATCTTCATGAGGTAGTCAG-3'.

### Data analysis

Statistical difference were evaluated using ANOVA with Dunnett's *post hoc *test and considered significant at *P *< 0.05.

## Results

### Effect of bortezomib on ROS generation in thyroid cancer cells

**Figure 1:**
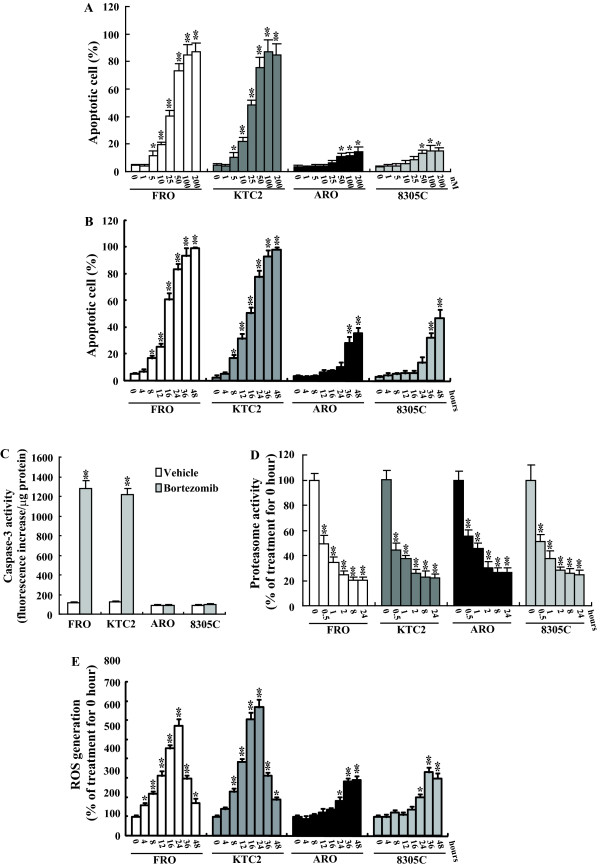
**Generation of ROS during bortezomib treatment**. A, Cells were treated with the indicated concentration of bortezomib for 24 hours and apoptotic cells were analyzed. B, Cells were treated with 50 nM bortezomib for the indicated time, apoptotic cells were analyzed using annexin V/PI double staining and subsequent flow cytometry. C, Cells were treated with 50 nM bortezomib for 8 hours and intracellular caspase 3 activity was measured. D-E, Cells were treated with 50 nM bortezomib for the indicated time, proteasome activity (D) and production of ROS (E) were analyzed. *, *P*< 0.05; ** *P *< 0.001 *vs *vehicle (A, C) or 0 hour treatment (B, D-E).

In our recent studies, we had demonstrated that proteasome inhibitors exhibited significant cytotoxicity against human undifferentiated thyroid cancer cells [22]. Consistent with our previous report [22], FRO and KTC2 cells were sensitive to bortezomib treatment with IC_50 _values in the range of 25–50 nM (Figure 1A). ARO and 8305C cells demonstrated limited cell toxicity, less than 20% cell death was observed even in the presence of high concentration of bortezomib (Figure 1A). Bortezomib was a potent death inducer in FRO and KTC2 cells, fewer cells were alive after treatment for 36–48 hours (Figure 1B). On the other hand, Bortezomib-induced apoptotic death was delayed in ARO or 8305C cells. Prior to treatment for 24 hours, little cell death was observed in these tumor cells. Moreover, almost half of cells survived even after exposure for 48 hours (Figure 1B). The cell death effects of bortezomib in thyroid cancer cells were confirmed by caspase-3 activity assay (Figure 1C). Bortezomib quickly suppressed proteasome activity, about 50% suppression was observed upon exposure to bortezomib for half hour (Figure 1D). The suppression peaked at 2 hours and remained within 24 hours (Figure 1D). Bortezomib induced almost equal suppression in all these thyroid cancer cells (Figure 1D), suggesting that inhibition of proteasome activity *per se *is not sufficient to induce cell death. We then investigated whether proteasome inhibition affect the generation of ROS in thyroid cancer cells. Exposure to bortezomib caused an increase in ROS levels in FRO thyroid cancer cells as measured by DCF fluorescence. Without bortezomib treatment, there were no significant differences in ROS levels among these thyroid cancer cells (data not shown). The relative levels of ROS were elevated as early as 4–8 hours after exposure to bortezomib, peaked at 24 hours and gradually declined in FRO cells (Figure 1E). Similar results were also observed in KTC2 cells (Figure 1E), which also demonstrated very sensitive to bortezomib-induced apoptosis. ARO and 8305C cells were relatively refractory to bortezomib-mediated ROS release (Figure 1E). In these tumor cells, no significant DCF fluorescence was detected prior to 8–12 hours and ROS production was visualized 24–36 hours post-bortezomib treatment (Figure 1E). Maintaining low levels of ROS in response to bortezomib until late times after treatment, suggesting that bortezomib was not an effective inducer of ROS production or elevation of intracellular antioxidants quickly scavenged the ROS in these tumor cells.

### Effects of bortezomib on intracellular glutathione level

**Figure 2:**
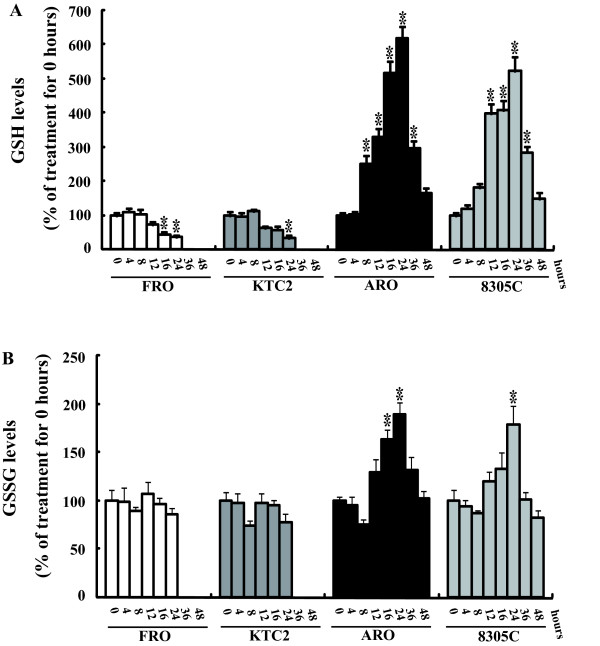
**Alteration of glutathione upon bortezomib exposure**. Cells were treated with 50 nM bortezomib for the indicated time, intracellular GSH (A) and GSSG (B) levels were measured. ** *P *< 0.001 *vs *0 hour treatment.

Distinct ROS generation patterns prompted us to compare the intracellular levels of the reducing agent GSH, the most abundant intracellular antioxidant, in thyroid cancer cells. Without bortezomib treatment, there were no significant differences in GSH levels among the panel of thyroid cancer cells (30–40 nmol/mg protein). With bortezomib treatment, no obvious alterations in GSH levels were observed in FRO and KTC2 cells at 4–8 hours, a significant fall was demonstrated starting at 12 hours after exposure (Figure 2A). ARO and 8305C cells had levels of GSH that were even higher than vehicle-treated controls at 8 hours after exposure. The amount of reduced GSH was further increased at 12–16 hours, and peaked at about 24 hours after exposure (Figure 2A). Since the increase in reduced GSH could be derived from reduction of oxidized glutathione (GSSG) by glutathione reductase or from *de novo *synthesis [23], we investigated the amount of GSSG upon exposure to proteasome. No marked alteration in GSSG amount was observed in FRO and KTC2 cells within 24 hours (Figure 2B). In ARO and 8305C cells, the amount of GSSG was even elevated at 16–24 hours of bortezomib exposure and restored to the basal level after 36 hours (Figure 2B). As the amount of GSSG was not decreased, but even elevated at some time points in ARO and 8305C cells, it is suggested that reduction of GSSG was not responsible for GSH elevation in these cells.

### Bortezomib induces expression of GCLC, the catalytic subunit of γ-GCS

**Figure 3:**
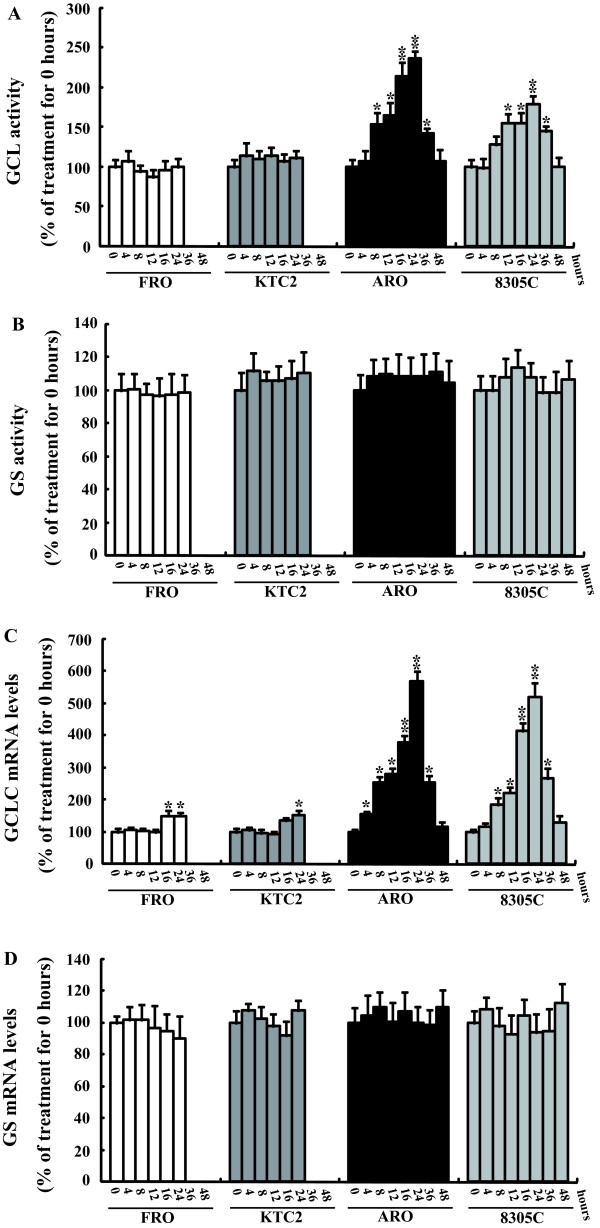
**Effects of bortezomib on the synthesis of glutathione**. Cells were treated with 50 nM bortezomib for the indicated time, intracellular GCL (A) and GS (B) activity were measured. In the parallel experiments, GCLC (C) and GS (D) mRNA levels were investigated using real time PCR. *, *P*< 0.05; ** *P *< 0.001 *vs *0 hour treatment.

GSH is synthesized by glutamate cysteine ligase (GCL) and glutathione synthetase (GS). GCL is also known as γ-glutamylcysteine synthetase (γ-GCS), a rate-limiting enzyme in glutathione synthesis. To determine the molecular details of glutathione elevation upon bortezomib treatment, we went on to assay the activity of two GSH synthetic enzymes. Alteration of GCL enzymatic activity was not observed in FRO or KTC2 cells (Figure 3A). However, GCL enzymatic activity was significantly increased in ARO or 8305C cells at 8 hours and after exposure to bortezomib, and peaked at 16–24 hours (Figure 3A). On the other hand, GS enzymatic activity was not changed in any cells (Figure 3B). Furthermore, after treatment with bortezomib, GCLC (the catalytic subunit for GCL) mRNA expression was significantly increased at 4–8 hours after treatment with bortezomib in ARO and 8305C cells (Figure 3C). The levels of mRNA for GCLC were also slightly increased in FRO and KTC2 cells at 16–24 hours, but to a much lesser extent than in ARO or 8305C cells (Figure 3C). The GS mRNA levels were not changed after bortezomib treatment in any treatment group (Figure 3D). Because GCL is the rate-limiting enzyme in GSH synthesis, thus increased GCLC transcription and GCL enzymatic activity might be responsible for the increased synthesis of GSH in these tumor cells.

### Effects of antioxidants on bortezomib-induced apoptotic death in thyroid cancer cells

**Figure 4:**
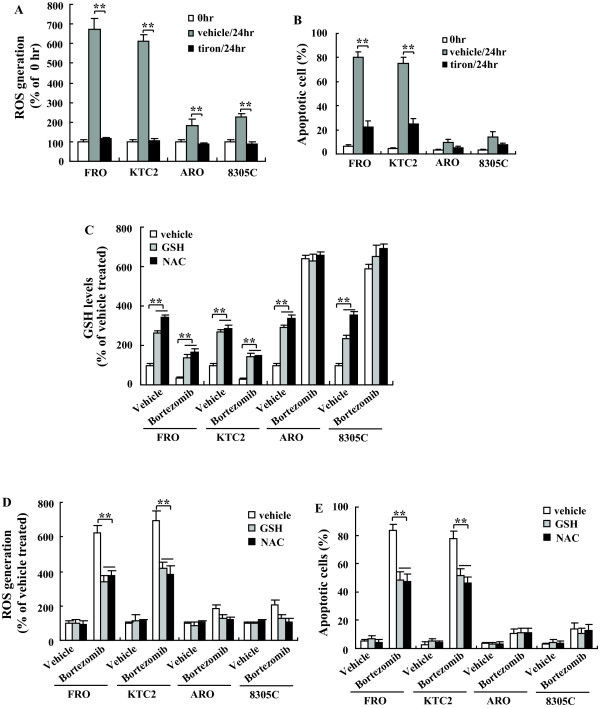
**Effects of antioxidants on the bortezomib-induced cell death**. Cells were treated with 50 nM bortezomib together with or without tiron for 24 hours, and production of ROS (A) and apoptotic cell (B) were analyzed. C, cells were pretreated with vehicle, GSH or NAC, sequentially treated with 50 nM bortezomib for additional 24 hours, and then intracellular GSH levels were measured. D, cells were treated as in C, and production of ROS was investigated. E, cells were treated as in C, and apoptotic cells were analyzed. *, *P*< 0.05; ** *P *< 0.001.

Because bortezomib treatment led to the enhancement of ROS generation prior to obvious apoptotic effects, it is possible that alterations in the cellular superoxidant state could play a role in the sensitivity to bortezomib-induced apoptosis. To examine whether elevated ROS is crucial for bortezomib-induced apoptosis in thyroid cancer cells, the cell-permeable superoxide scavenger tiron was utilized to perturb the bortezomib-induced ROS generation and then to examine the consequent events. Cotreatment with 1 mM tiron for 24 hours resulted in the complete abrogation of bortezomib-induced generation of ROS (Figure 4A). Abolishment of intracellular superoxide by tiron provided significant protection against apoptosis caused by bortezomib (Figure 4B). To clarify the contribution of intracellular glutathione to ROS suppression, we examined the effect of pretreatment with GSH, or N-acetyl-L-cysteine (NAC), which is a precursor of glutathione and elevates glutathione contents. Glutathione content was significantly elevated by treatment with 10 mM GSH for 4 hours or 10 mM NAC for 24 hours (Figure 4C). Pretreatment with GSH or NAC abrogated the decrease in intracellular GSH level mediated by bortezomib in FRO and KTC2 cells (Figure 4C). Bortezomib alone significantly increased the intracellular GSH in ARO and 8305C cells, whereas GSH or NAC pretreatment had little effect (Figure 4C). Pretreatment with GSH or NAC significantly suppressed bortezomib-induced ROS generation (Figure 4D) and provided significant protection against apoptosis caused by bortezomib in FRO and KTC2 cells (Figure 4E). Minor apoptotic cells were observed in ARO and 8305C cells, and GSH or NAC pretreatment did not provide further protection (Figure 4E).

### Depletion of reduced glutathione level sensitizes thyroid cancer cells towards bortezomib-mediated apoptosis

**Figure 5:**
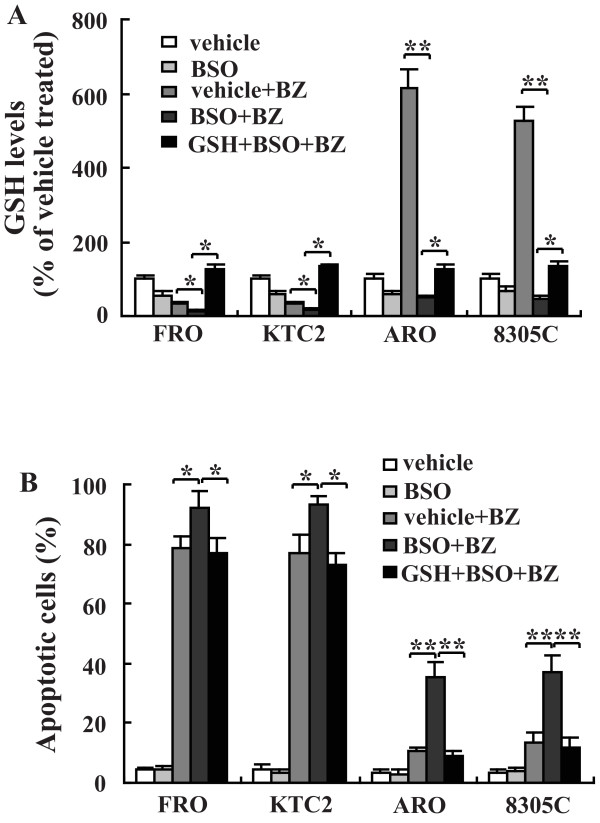
**Effects of intracellular GSH on the bortezomib-induced cell death**. Cells were pretreated with BSO alone or BSO and GSH monoethyl ester, 24 hours later after sequential treatment with vehicle or bortezomib, intracellular GSH level (A) and apoptotic cells (B) were analyzed. *, *P*< 0.05; ** *P *< 0.001.

To further explore whether modulation of GSH level would modify the cell response to apoptosis induced by bortezomib, buthionine sulfoximine (BSO), an irreversible inhibitor of GCL was incubated prior to bortezomib treatment. Following a 4-hour treatment with 0.2 mM BSO, the intracellular GSH level was markedly reduced (Figure 5A). BSO treatment increased apoptosis induction both in sensitive and insensitive cells (Figure 5B). 10 mM GSH monoethyl ester was further utilized to replete with intracellular GSH level after BSO treatment. Following a 3-hour GSH monoethyl ester treatment, an -2 to 3-fold increase in intracellular GSH level was detected (Figure 5A). When cells were supplement with exogenous GSH monoethyl ester with BSO also present, GSH monoethyl ester treatment reversed the effect of BSO on apoptosis induced by proteasome inhibition (Figure 5B).

## Discussion

The proteasome is responsible for the degradation of most cellular proteins and is therefore critical to the regulation of many processes, including cell cycle progression and apoptosis. The role of the proteasome in regulating the growth and survival of tumor cells makes it an attractive therapeutic target. Proteasome inhibitors have emerged as a new group of chemotherapeutic agents for human cancer[1]. Bortezomib is a small molecule proteasome inhibitor that has shown antitumoral activity in a number of cell types and is currently undergoing clinical trials. The mechanisms underlying antitumoral effects of bortezomib are not totally defined. In the present work, we show that ROS generation is implicated in bortezomib-induced thyroid cancer cell death. The inhibition of ROS significantly suppressed bortezomib-induced apoptosis in sensitive cells. In support of the role of ROS in bortezomib-induced apoptosis, we found that ROS were transiently and weakly induced in less sensitive cells. Furthermore, depletion of intracellular GSH sensitized thyroid cancer cells to bortezomib-induced apoptosis, whereas repletion of GSH inhibited bortezomib-mediated thyroid cancer cell death.

ROS are now thought to be involved in several cellular mechanisms including apoptosis [24]. Studies in a variety of tumor cell types have suggested that cancer chemotherapy drugs induce apoptosis in part by generating endogenous oxidants [25]. Increasing evidence now demonstrates that the redox status of cells is an important factor in determining whether tumor cells can withstand chemotherapy [26]. Although there are multiple potential detoxification mechanisms that affect the efficacy of chemotherapeutic drugs and confer drug resistance to targeted cells, GSH has a prominent role in resistance to chemotherapy [27]. GSH and its associated enzymes play a critical role in the cell susceptibility to the cytotoxic effect of alkylating agents, doxorubicin, cisplatin and nitrosoureas [28]. It has been shown that for these drugs, increased cellular GSH levels can confer cells resistance and decreased cellular GSH levels can sensitize cells to the killing effects [29,30]. Interest for clinical trials in modulating GSH level to increase tumor cell response to anticancer drug treatment is still growing [31,32].

Generation of ROS is now considered to be the early and critical events for the initiation of bortezomib-induced apoptotic signaling in some human cancer cell lines [5,11-13]. It has also been reported that GSH could not inhibit bortezomib-induced cell death in some lung cancer cell lines [33]. In this study, we propose that production of ROS is associated with bortezomib-induced apoptosis in thyroid cancer cells. Although the reported results to date may seem contradictory, they may more likely represent tissue and cell type-specific differences. Our results indicate that bortezomib induces the production of oxygen species, which participate in signaling, intracellular anti-oxidant defense systems to quench some of these ROS, reducing bortezomib-induced apoptotic signaling and therefore drastically reducing the frequency of apoptosis. GSH is the most abundant cellular thiol and plays a central role in maintaining cellular redox status and increases in GSH levels would be expected to reduce ROS levels and antagonize apoptotic signals [34]. In the current study, we found that bortezomib induced quick increase in ROS production, and subsequent decrease in GSH levels in sensitive thyroid cancer FRO and KTC2 cells. On the other hand, in insensitive thyroid ARO and 8305C cells, bortezomib had little effect on ROS generation and increased the intracellular GSH level within 24 hours. During this period, it is intriguing that bortezomib induced upregulation of GCLC and activation of GCL in ARO and 8305C cells, whereas had little effect in FRO and KTC2 cells. Although the thyroid origin of ARO cells has been recently questioned [35], our results suggest that ROS generation might be implicated in the cytotoxic response of some cancer cells to bortezomib and ability of cancer cells to induce GCL and subsequent GSH levels, thereby scavenging ROS is, at least in part, involved the cytotoxic responses to bortezomib exposure.

## Conclusion

ROS generation is implicated in cytotoxic responses of thyroid cancer cells to bortezomib. Sensitivity of thyroid cancer cells to bortezomib is associated with the inability to induce GSH synthesis and subsequent GSH levels, thereby quenching ROS.

## Competing interests

The authors declare that they have no competing interests.

## Authors' contributions

ZXD carried out the molecular genetic studies, cell culture, participated in the data analysis and drafted the manuscript. HYZ carried out the immunoassays. XM participated in immunoassays and cell culture. YG participated in the manuscript drafting. HQW conceived of the study, and participated in its design and coordination. All authors read and approved the final manuscript.

## Pre-publication history

The pre-publication history for this paper can be accessed here:

http://www.biomedcentral.com/1471-2407/9/56/prepub
